# Coproducing multilingual conversational scripts for a mental wellbeing chatbot - where healthcare domain experts become chatbot designers

**DOI:** 10.1192/j.eurpsy.2022.748

**Published:** 2022-09-01

**Authors:** H. Nieminen, L. Kuosmanen, R. Bond, A.-K. Vartiainen, M. Mulvenna, C. Potts, C. Kostenius

**Affiliations:** 1 University of Eastern Finland, Department Of Nursing Science, Kuopio, Finland; 2 Ulster University, School Of Computing, Belfast, United Kingdom; 3 University of Eastern Finland, Department Of Health And Social Management, Kuopio, Finland; 4 Luleå University of Technology, Department Of Health, Education And Technology, Luleå, Sweden

**Keywords:** development process, chatbots, mental wellbeing, digital interventions

## Abstract

**Introduction:**

Digital mental health interventions, such as chatbots that promote mental health and wellbeing are a promising way to deliver low-threshold support 24/7 for those in need. According to current knowledge about the topic, health care professionals should participate in the design and development processes for digital interventions.

**Objectives:**

The aim of this presentation is to describe the interdisciplinary content development process of the ChatPal chatbot.

**Methods:**

The content development process started in co-operation with mental health professionals and potential users to identify requirements. Content was created, evaluated and tested in international, multi-disciplinary group workshops, and online tools were used to allow the collaboration. Initial conversational scripts were drafted in English, and translated into Finnish, Swedish and Scottish Gaelic.

**Results:**

A multilingual chatbot was developed and the conversation scripts were structured and stored using a spreadsheet. The conversation scripts will be made freely available online in due course using this structured approach to formatting chatbot dialogue content. It will allow repurposing the content as well as facilitating studies that wish to assess the design of conversation scripts for mental health chatbots. Conversation design process also highlighted some challenges in turning empathetic and supportive conversations to short utterances suitable for a chatbot.

**Conclusions:**

The ChatPal chatbot is now available in four languages. As literature about the topic is still scarce, it is important to describe and document the content development processes of mental health chatbots. Future work will develop a conversational UX toolkit that would allow health professionals to design chatbot scripts using design guidelines.

**Disclosure:**

No significant relationships.

